# Clustering and spatial heterogeneity of bovine tuberculosis at the livestock/wildlife interface areas in Namwala District of Zambia

**DOI:** 10.14202/vetworld.2020.478-488

**Published:** 2020-03-14

**Authors:** Novan Fully Proud Tembo, John Bwalya Muma, Bernard Hang’ombe, Musso Munyeme

**Affiliations:** 1 Department of Public Health, School of Health Sciences, University of Lusaka, Lusaka, Zambia; 2 Department of Disease Control, School of Veterinary Medicine, University of Zambia, Lusaka 10101, Zambia; 3 Department of Paraclinical Studies, School of Veterinary Medicine, University of Zambia, Lusaka 10101, Zambia

**Keywords:** geographic information system, reservoir hosts, spatial distribution, zoonotic tuberculosis (bovine tuberculosis)

## Abstract

**Background and Aim:**

Bovine tuberculosis (bTB) remains a major public health issue in Zambia and has been exacerbated by human immunodeficiency virus prevalence and consumption of unpasteurized milk in the Southern Province of the country. The prevalence of bTB has been established to be linked to Kafue Lechwe, which act as reservoir hosts and share grazing fields with domestic cattle. No studies have so far used geographic information system (GIS) to investigate the relationship between the reservoir hosts (Kafue Lechwe) and domestic animals. This study, therefore, aimed to apply GIS to investigate the spatial distribution of bTB in Namwala District of the Southern Province of the country.

**Materials and Methods:**

To investigate the spatial distribution of bTB, geographical positioning system (GPS) coordinates representing 96 cattle herds across 20 independent villages were captured alongside risk factor data. The 96 herds were based on abattoir reports of condemned carcasses and a trace back. Positive herds were confirmed by cross-reference to purified protein derivative tests conducted by the District Veterinary Office. The GPS coordinates were transferred into ArcView 3.2 and laid on the map of Namwala District alongside physical features, including national parks, game management areas, and flood plains. Questionnaires were administered across 96 independent households to assess risk factors of bTB transmission.

**Results:**

The results revealed a “clustered” spatial distribution of the disease in cattle in Namwala District of Zambia, particularly significant in the eastern interface areas of the district (p=0.006 using Moran’s I). Abattoir to production area trace back revealed a herd-level prevalence of 36.4% (95% CI=26.7-46.3%) among cattle herds in Namwala District, whereas individual animal prevalence ranged from 0% to 14% (95% CI=2.4-26.2%). Further, GPS data indicated that the majority of the positive herds were located at the livestock/wildlife interface area. Contacts with wildlife, coupled with sharing grazing, and watering points were found to be significant risk factors for bTB transmission.

**Conclusion:**

This study demonstrated the presence of bTB in cattle and associated spatial risk factors. In particular, bTB was observed to be a function of animal location within the livestock/wildlife interface area. GIS is thus an applicable and important tool in studying disease distribution.

## Introduction

Bovine tuberculosis (bTB) caused by *Mycobacterium bovis* is a major zoonotic disease of worldwide economic and public health importance, especially in developing countries where control measures are neither formulated nor enforced [1,2]. In fact, bTB belongs to closely related bacteria classified under members of the *Mycobacterium tuberculosis* complex (MTC). Notably, bTB has been shown to exhibit a wide host range and preference than most pathogens within the MTC, making it a very important emerging zoonotic pathogen [1]. Among the MTC, *M. tuberculosis* is the most common cause of human TB, but an unknown proportion of cases are due to *M. bovis* [3,4]. The relatively low incidence of the development of open (infectious) pulmonary TB due to bTB in humans is almost certainly due to immunological factors that could be abrogated in dual HIV/AIDS infections [1,2]. Thus, there is every reason to be seriously concerned that the HIV pandemic might fuel an increase in human TB due to bTB [4-6]. The World Health Organization has declared the new resurgence of TB a global emergency. Globally, approximately 1.86 billion people are infected with TB (henceforth to refer to both *M. bovis* and *M. tuberculosis* originating types), implicating TB as the greatest cause of death due to a single pathogen, second only to the HIV [7-9]. In some developed countries, bTB has been brought under control, with a subsequent decrease in economic losses attributable to it [10,11]. A consequence of the reduced burden of infections in animals in these countries is a reduction in human TB cases [12]. However, the situation is different in most developing countries such as Zambia, where bTB is still endemic. Currently, the global incidence rate is growing at approximately 0.4% per year, with a much higher rate observed in sub-Saharan countries, Zambia included [12].

Geovisualization or geographic information system (GIS) mapping is the use of computer-assisted graphical methods to visualize geospatial information. It is a method that is used to help guide and direct health service planning, public health interventions and to capture and reveal disease hotspots [13]. The aim is not always to establish statistically significant relationships, but rather to have a way to understand the ways in which health status depends on space (environment) and to bring out information on the probable factors behind this variation. Such aims are informed by the realization that poor or non-user-friendly presentation of results hampers mitigation efforts [14]. Understanding the distribution of a disease is important in the formulation of control strategies. Moreover, the distribution is also important in the appreciation of the extent of disease prevalence. Although there are a number of reports on bTB infections in cattle, there are only a few reports about the disease distribution [13,14]. Up-to-date information on the prevalence in relation to spatial distribution of bTB in animals is required in the light of high HIV/AIDS prevalence in sub-Saharan Africa [15-17]. Moreover, animal protein (meat and milk) is highly required to mitigate the impact of the HIV/AIDS pandemic, but bTB infections threaten this resource [18]. The spatial modeling available using GIS can be used to understand the spatial variation of disease with respect to environmental factors and health-care systems. GIS can assist more importantly in this field by filling the gaps through practical disease modeling techniques [13,16]. Therefore, GIS can be used to model TB (both bTB and human type) in Zambia, which is one of the leading causes of death worldwide, killing more people aged over 5 years of age along with AIDS, malaria, and diarrhea. However, before modeling, it is important to get the pattern of the disease using mapping [14,16]. Like other technology-intensive endeavors, the use of GIS is still limited in Africa such that it is not clear how well it can be used and sustained in an African setting while it has been tried and its limitations and strengths have already been established in the developed world [17-23].

Based on the literature reviewed, a number of studies have been done on TB in Zambia. However, none has focused on spatial distribution, let alone the use of GIS tools in relation to risk factors. Such information would be vital to the management of the disease. If control programs were to be implemented with the available information, it may be difficult to know exactly where those efforts would have to be directed. The aim of this study was, therefore, to use GIS to help identify the spatial distribution of bTB in cattle of Namwala District of Zambia and identify spatially associated risk factors.

## Materials and Methods

### Ethical approval and informed consent

The study did not require ethical approval as advised by the Department of Disease Control, School of Veterinary Medicine of the University of Zambia under which the study was conducted. However, informed consent was still obtained from each participant and confidentiality was observed.

### Study areas

**Figure-1:**
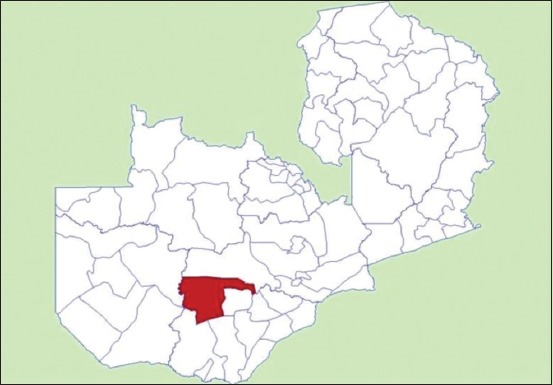
Map of Zambia, showing the geographical location of Namwala District in relation to other Districts in Zambia.

The study was conducted in Namwala District of Zambia located at 15°45’S and 26 °27’E (Figure-1).

**Figure-2:**
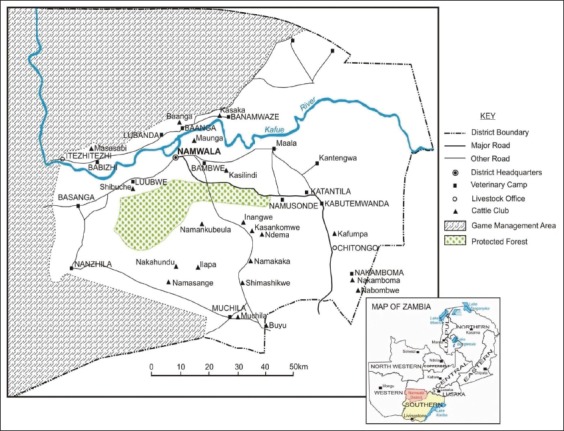
Detailed Map of Namwala District, relative to the game management area, showing the names of some of the villages and areas under study, insert shows the Map of Zambia (Munyeme, 2010; co-author).

This area was selected because it is the main cattle producing area in Zambia and previous reports had intimated the existence of high bTB prevalence [24]. Further, the people in this area have a tradition of drinking raw milk which has been considered a risk factor for exposure to *M. bovis*. The study was conducted from October 2012 to March 2013 across different villages represented under the constituencies and cattle clubs therein (Figure-2).

### Study design

The study was conducted as a cross-sectional study. The two major slaughterhouses in Namwala were included to obtain daily bTB condemnation rates. Both retrospective and prospective data were collected to check on the origin of the bTB infected cattle. From abattoir condemnations reports of suspected TB infected cattle, a trace back was made to the areas of origin to obtain GIS information and administer questionnaires. A positive herd was defined as one from which an animal was condemned at the abattoir for suspected TB lesions and clinical investigation of the herd, and interviews with the owner showed that there was still at least one animal showing clinical signs characteristic of bTB such as coughing and emaciation. Confirmation was done with reference to secondary data as recorded by animal health inspectors from the district veterinary department who were certifying the bTB status of the cattle herds using the purified protein derivative tuberculin skin test as required by the Government of the Republic of Zambia.

### Abattoir studies

**Figure-3:**
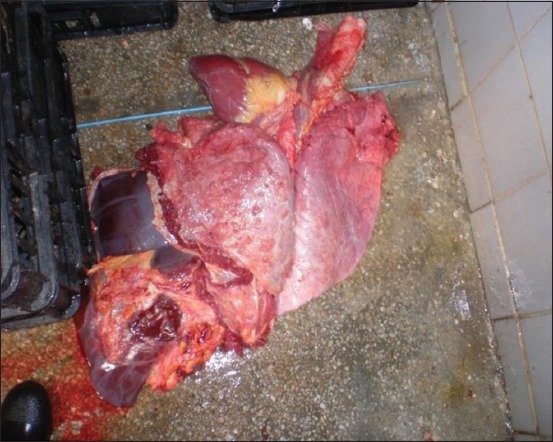
Condemned lungs due to suspected infection with bovine tuberculosis (see the arrows). The criterion was the caseous material and tubercles in the pathological lungs.

The study aimed to map the spatial distribution of the bTB cases as determined at the two abattoirs. The initial task was thus to collect data from daily condemnation reports at the abattoirs. A presumptive positive case of TB was defined as carcass in which tuberculous lesions were observed at postmortem inspection. The diseased lungs were those containing tubercles and caseous material (Figure-3).

The postmortem findings were correlated with clinical findings such as emaciation and coughing of the animals. The points of origin of the positive individual animals were then geo-referenced. Interviews were also conducted in the households owning the respective herds from which the abattoir condemned positive cases originated. The follow-up was made possible by the records of the owners as compiled by the abattoirs. The follow-up was to link the positive cases to the herds and thus map the spatial distribution of the positive bTB cases. Cross-reference was also done to test results as recorded by the District Veterinary Office for positive herds based on the tuberculin test.

### Questionnaire survey

The survey was done using purposive sampling. The questionnaire survey was conducted among the households whose herds had a positive animal condemned as being bTB positive on postmortem at the abattoirs and confirmed as such by animal health workers. The survey was to understand the risk factor that may be contributing to the disease transmission and to determine positive herds in relation to positive cases by interviewing herd owners on relevant clinical signs of bTB. A closed-ended pre-tested structured questionnaire was used to collect data on age, sex, grazing behavior, drinking water sources, and other production factors of cattle reared. Demographic characteristics were to be collected to help policy-makers structure intervention strategies by looking at characteristics of the target population.

#### Collection of GIS data

A trace back system was employed where geographical points were collected from points of origin of the carcasses deemed positive for TB on postmortem examination. The geographical points were captured using a geographical positioning system (GPS) device and recorded.

#### Collection of epidemiological data amenable to GIS

A closed-ended pre-tested structured questionnaire was used to collect data on age, sex, and other production factors of cattle reared. The biodata of the cattle rearing communities was also collected and accompanied with demographical and ecological data and from this information descriptive statistical tests were run.

### Data analysis

#### Geospatial data

The collected coordinates were processed and transferred onto maps of Namwala to show bTB occurrence. The general pattern helped to determine how bTB distribution relates to the interface area. The processing and creation of maps were done in Arc-GIS 3.2. The points were first transferred onto a Microsoft Excel 2007 spreadsheet for cleaning and conversion to wholly decimal coordinates since the GPS device captured was in the form of ‘degrees’ and ‘minutes’. The cleaned data were stored as a text file, which were transferred into ArcGIS 3.2 to lay on the processed maps of Namwala District. The map layering included a combination of shapefiles to show important features of the district including most importantly, the flood plains, the rivers, game management areas (GMAs), and national parks. The captured coordinates were laid on the created maps to gain an understanding of how they relate to those important geographical features such as the flood plains, the GMAs, and national parks. The GMAs and national parks have been known to play a role in being the basis for the interface of wildlife and domestic animals where transmission of bTB has been known to occur. The analysis attempted to show how the coordinates indicating where animals found positive for TB originated related to the interface, which was a risk factor for the disease. While transmission occurs between livestock and wild animals, it does not always involve livestock wandering into GMA’s or national parks and neither does it always involve wild animals wandering far into grazing areas. Mixing tends to occur in areas that lie between either GMA’s or national parks and grazing area (interface). For our analysis, we posited buffer zones as interface areas where transmission of TB may occur from wild animals to livestock.

Based on findings from a study in Nepal using GIS investigating buffer zones, 10-30 km buffers were created [25] from the parks (and GMA’s), which actually covered most points of origin of the bTB-positive animals. To gain more insight into the relationship of bTB and the interface, shapefiles of the GMAs were also added to the maps, which completely captured the collected coordinates indicating the relationship between the two. To further understand how significant the coordinates were in terms of following geographical location, we employed Moran’s I to test significance played by location.

### Descriptive statistics

The database establishment was in Microsoft Excel^®^ 2007 before transferring to Stata SE12 for Windows (Stata Corp. College Station, TX) for further analysis. The database included biodata, bTB test results as well as other epidemiological data. The proportion of bTB infected cattle in those slaughtered at the two slaughterhouses was estimated. Extraneous factors that could explain the infection of bTB in humans were described using both qualitative and analytical tools. The questionnaire was used to investigate possible factors that lead to domestic animals contracting bTB from reservoir hosts and possible practices leading to disease in humans. To assess the plausibility of the factors captured in the questionnaire, univariate analysis was used. Moreover, to understand the interaction of the factors observed on univariate analysis multiple regressions was used. Descriptive statistics were used to highlight characteristics of at-risk population and to help policy-makers in formulating control measures such as being able to know the most affected age group or sex group.

## Results

### Descriptive results

Using trace back system based on abattoir survey reports, a total of 96 herds of cattle from 20 different geo-captured villages in Namwala District were sampled for bTB positivity. The herd positivity was based on condemnation at the abattoirs and further information on relevant clinical signs gathered during interviews after tracing back to villages of origin of the individual animals condemned. Confirmation was from data records from animal health inspectors who carry out bTB testing using the purified protein derivative. From these, a total of 35 herds were found positive representing a herd-level prevalence of 36.4% (95% CI=26.7-46.3%) (Table-1). Individual animal prevalence ranged from 0% to 14% (95%=2.4-26.2%) across the study areas. Out of the 96 herds of cattle, 7 (7.3%, 95% CI; 2.0-12.6%) practiced sedentary/village grazing the practice of not taking cattle to the plains, while 87 (90.6%, 95% CI; 84-96.5%) practiced transhumance cattle grazing system representing of the herds in the area. Only two herds (2.1%, 95% CI; 0-4.9%) of the 96 sampled herds were kept in the plains almost permanently. There were more positive herds in the Eastern part of Namwala District toward Lochinvar National Park, and this area formed the biggest interface area between cattle and the Kafue Lechwe antelopes. The grazing system or cattle keeping enterprises ranged from farmers practicing plain grazing (grazing in the flood plains) in the lacustrine flood plains of the GMAs, to those that practiced transhumance and those that had sedentary herds which were always in the villages.

**Table-1:** The individual prevalence of bovine tuberculosis from the 20 locations geo-captured.

Location No. herd	Number of herds	Area prevalence %	95% CI
Namwala central	21	14	2.4-26.2
Chinyemu	18	0	n/a
Baambwe	25	8.6	0-18
Kawalizhi	22	11.4	0.5-22.3
Musongwa	19	2.8	0-8.5
Mbeza	90	5.7	0-13.6
Mbeza	89	5.7	0-13.6
Kwa Nico	71	8.6	0-18.1
Chitongo	95	2.9	0-8.5
Maala	12	2.9	0-8.5
Maala	29	5.7	0-13.6
Nakwanza	13	8.6	0-18.1
Kantengwa	14	2.9	0-8.5
Mahungu	19	2.9	0-8.5
Butele	175	5.7	0-13.6
Kabulamwanda	205	2.9	0-8.5
Kasaka	130	0	n/a
Kasenga	106	0	n/a
Shikolopa	100	0	n/a
Shimbizi	177	8.5	0-18.1

**Figure-4:**
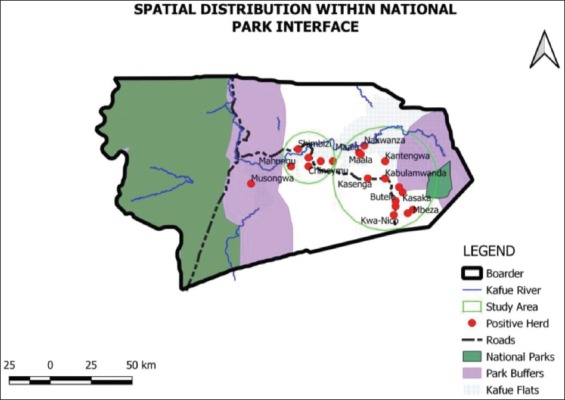
Spatial distribution of villages (dots), which had positive cattle herds in proximity to interface areas and National Parks, circles within the figure indicate the approximate geographical distribution in relation to the size of cattle herds being positive.

The cluster of villages studied, grouped under one village name, had in the previous 12 months under study at least had one TB patient who had been treated or was receiving treatment for TB, according to the information gathered during the interviews. All these studied areas had their GPS location collected (Figure-4).

The smaller circle within Figure-4 indicates mainly cattle herds around Namwala Central, Baambwe areas and those near or in proximity to Maala region.

Majority of cattle herds that were found to be positive belonged to the villages in the eastern part of Namwala District 70% (n=14 villages), with 50% (n=10 villages) being located deep inside the buffer zone (the GMA) between the open areas and the National Park as indicated by the larger circle.

### Prevalence by strata

There was an overall significant difference in bTB prevalence across the 20 villages studied based on individual animal data (p=0.001). Based on results for condemnation of carcasses, the individual herd prevalence of bTB ranged from 0 to 14%. In most areas, the prevalence oscillated around 3% (Table-1).

**Figure-5:**
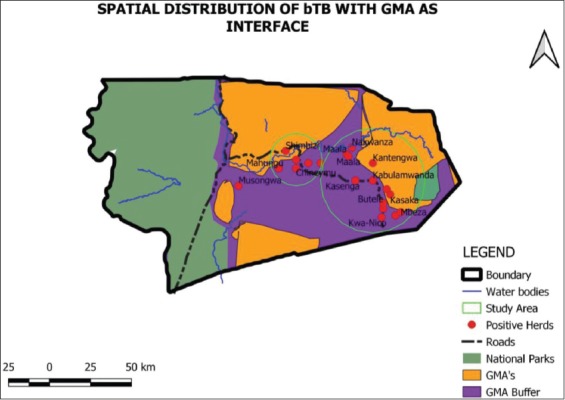
The bovine tuberculosis cases within a 10 km buffer of the game management areas.

When GMAs were considered, the positive herds still tended to fall within the areas where mixing with wildlife occurs. The positive herds were within a 10 km buffer of the management areas (Figure-5).

To test for correlation of positive bTB herds in the observed geographical areas, Moran’s I was used, which indicated a considerable correlation with p=0.006 (Table-2). The correlation measures the tendency to occur in a given geographical area.

**Table-2:** The test for autocorrelation was done using Moran’s I.

Variables	Moran’s I			Z	p-value*

	I	E(I)	Sd (I)		
Herd size *1-tail test	0.18	−0.053	0.093	2.519	0.006

* p≤0.05

### Demographic characteristics of study populations

A total of 20 village clusters were studied from within the main villages, there were about 3-6 independent villages that owned cattle separately, and these formed 96 respondents who correspondingly had 96 cattle herds. A range of biologically plausible risk and deterministic factors of disease occurrence were studied and carefully examined their effect within the study populations and are initially expressed as proportions (Table-3).

**Table-3:** Summary of biologically plausible deterministic and risk factors that were considered across all the study villages and their proportional representation.

Risk factor	Proportion %	Confidence interval
Positive herd	36.46	26.7-46.30
Bacille Calmette-Guérin vaccination	29.17	19.9-38.4
Treated for TB	65.63	56.0-75.3
TB knowledge	40.63	30.6-50.6
Sour milk drinking	79.2	70.9-87.4
Boiling milk	26.04	17.1-35.0
Freq. of milk	81.25	78.0-97.8
Keeping cattle	97.95	93.3-99.7
Condemnation	66.67	57.1-76.3
Drinking water sharing	91.67	86.0-97.3
Contact with wildlife	52.08	41.9-62.3

TB=Tuberculosis

Demographic characteristics of the respondents were as follows: Gender and female-headed households represented (1%, 95% CI; 0-3.1%), while male-headed households represented (99%, 95% CI; 96.9-100%) of the respondents. Similarly, 70% of the respondents were living within the buffer zones of the National Parks, with close to 60% coming from the eastern GMAs close to Lochinvar National park. The minimum number of people living in a household was 2, while the maximum number was 39.

### Univariate analysis

When considered independently, some of the significant factors that showed high proportional representativeness as contributing to disease transmission and maintenance included low Bacille Calmette–Guérin (BCG) vaccination at 29%, (95% CI; 19.9-38.4) the low level of knowledge on bTB at only 41%, (95% CI; 30.6-50.6) the high frequency of drinking unpasteurized milk at 81%, and (95% CI; 78.0-97.8) with the frequency of drinking at least 3 times a week, being a maintenance factor. Other plausible factors included low number of those boiling their milk at only 26% (95% CI; 17.1-35.0) and the high contact between domestic animals and wild animals at water points at 92% (95% CI; 86.0-97.3) (Table-3).

On univariate analysis of independent variables at herd level, four main factors were observed to have had an independent effect on TB herd status (Table-4).

**Table-4:** Some risk factors which were considered significant at univariate analysis.

Risk factor	Chi-square	p-value
Contact with wildlife	9.42	0.002
Movement to the plains	10.12	0.001
Sharing drinking water with wildlife	5.62	0.017
Condemnation at abattoir due to tuberculosis	5.76	0.016

Variables with Fisher’s exact p≤0.25 were identified as risk factors for inclusion in the multivariable model (and all the above variables thus transferred for analysis in the multivariate analysis logistic regression model)

Some variables had marginal effects while others had no effect. Of these variables, the most significant were contact with wildlife, being transhumant, moving cattle herds to the plains (thus moving of cattle to the plains for grazing for certain parts of the year), and cattle sharing grazing ground and drinking water as well as having animals being condemned at the abattoir, these came out very prominent.

Contact with wildlife, when assessed independently, showed a significant effect in TB prevalence (Fisher’s exact *χ*^2^=9.42, p=0.002). Moving animals to the plains were noted to be significantly associated with TB positivity (*χ*^2^=10.12; p=0.001). Similarly, cattle herds that were seen to share drinking water and share grazing grounds had a significant effect on finding a herd positive within it (*χ*^2^=5.62; p=0.017). Cattle herds from which animals taken to the abattoir for slaughter, had at least one animal condemned within the preceding 12 months before the study period, showed a significant effect on TB positivity (*χ*^2^=5.76; p=0.016) (Table-4).

### Multivariate analysis of factors associated with bTB positivity in cattle

The multiple logistic regression model identified contact with wildlife, being transhumant (thus moving of cattle to the plains for grazing for certain parts of the year), cattle sharing grazing ground, and drinking water as well as having animals being condemned at the abattoir due to suspected TB in the herd as factors having significant effect on herd bTB status (Table-5). The Hosmer–Lemeshow goodness-of-fit check showed that the model fitted the data well with n=96, *χ*^2^ (6)=5.2 and p=0.52. Cattle herds that were reported to be sharing grazing grounds and drinking water with wild animals concurrently had a significant effect on bTB status (OR=5.5, 95% CI; 4.1-73.4) given that contact with wildlife and having animals being condemned at the abattoir due to suspected TB are controlled (Table-5).

**Table-5:** Herd-level risk factors for bTB positivity in cattle herds of Namwala District (n=96) in Zambia. Results from the final multivariable logistic regression model.

Variable	Level	*b*	SE(*b*)	p-value	OR	95% Confidence interval (OR)
Constant		1.3	1.1	0.19	-	-
Contact with wildlife	Yes	0.94	0.2	0.048	0.24	0.15-3.86
	No	-	-		1	-
Movement of cattle to the plains	Yes	1.2	1.1	0.09	0.28	0.32-2.40
	No	-	-	-	1	-
Sharing grazing and drinking water with wildlife	Yes	1.5	1.3	0.01	5.51	4.1-73.4
	No	-	-	-	1	-
Organs condemned at abattoir due to TB	Yes	0.5	0.4	0.04	1.1	0.29-1.3
	No	-	-	-	1	-

bTB=Bovine tuberculosis, TB=Tuberculosis

### Results from the final multivariate logistic regression model

Although there was no statistical difference between owners who had their cattle condemned at the abattoir due to suspected TB, or exhibiting TB lesions and those who had not (OR=1.1, 95% CI; 0.32-2.40), in general people who have had animals condemned at the abattoir tended to employ some precautionary measures or were known to be more responsive to bTB control programs because of the experience of losing out on having their animals condemned. Similarly, owners who had an experience of having their cattle condemned at the abattoir due to suspected TB, or exhibiting TB lesions, had a significant effect on bTB status given that contact with wildlife, being transhumant, and cattle sharing grazing ground and drinking water are controlled. Contact with wildlife as well as movement of cattle to the plains without actual sharing of drinking water and grazing pastures was found to have a protective effect on bTB herd status (OR=0.24, 95% CI; 0.153.86) and (OR=0.28, 95% CI; 0.32-2.40) given that the other two variables in the model are controlled.

## Discussion

The results obtained from this study starting from trace back system of suspected abattoir bTB-positive cattle and their geospatial referencing have revealed the spatial distribution of the disease in cattle in Namwala District of Zambia. The results have particularly revealed that there is an ecological interplay in the spatial distribution of bTB in the high bTB prevalence area of Namwala District, which is itself a function of the existence of the reservoir hosts particularly in the eastern interface areas of the district. These results are further congruent with earlier traditional epidemiological results of bTB being more or less proportionately high in areas with Kafue Lechwe antelope populations [24].

It is worth noting that bTB in wildlife forms a stable reservoir such that *M. bovis* has been characterized from Kafue Lechwe (*Kobus Leche Kafuensis*) [26]. The Kafue Lechwe is an effective disseminator of bTB more so with the habit of overcrowding, which promotes the sharing of the pathogen [27]. Where animals mix with Lechwe disease transmission have been observed to occur with a case of a Kafue Lechwe found dead with tubercles on a ranch where bTB was diagnosed in Central Province as one example. Kafue Lechwe has a role as a maintenance host and *M. bovis* is known to circulate in them. The prevalence is high in the interface area in Lochinvar National Park [27]. In fact, positive bTB was found in 14-35% of postmortems in Kafue Lechwe, confirming it as an important reservoir [28]. Moreover, Munyeme and Munang’andu [27] reported that 24% of the sampled Kafue Lechwe had gross lesions indicative of bTB from 2004 to 2008. The disease is also found in Wild Boars and African Buffaloes in South Africa, but in Zambia the Lechwe remains the important reservoir. Kafue Lechwe prefers swamps and lives in groups, which promotes disease propagation [28]. Flooding in the swampy areas also disseminates the infectious agent.

A lot of research has previously been done implicating the Kafue Lechwe in the sustenance and spread of bTB. With regard to spread to livestock, mixing in the plains is the contributing factor [24]. Moreover, older animals are more affected as well as those with poor body condition. The large herds in Namwala usually contain the two susceptible animal groups. Taken orally the infectious agent can be emitted as aerosols during rumination [27]. Aerosol transmission is more effective, while large doses are needed for oral transmission. Once in the livestock, the disease is spread from cattle to cattle. Animal movement on foot also sheds the disease in the routes of movement [29-31].

Despite these findings and earlier studies, bTB remains a huge problem in Namwala District of Zambia, as was indicated from abattoir results. Across the study District of Namwala, the disease burden at herd level was found to be as high as 36.4% (95% CI 26.7-46.3%). From the gathered trace back data obtained from affected herds, there were factors which were identified as relevant clinical signs of bTB including emaciation and coughing in relation to at least one animal from the herd that had at least one animal condemned as having tuberculous lesions on slaughter at the two abattoirs in Namwala District. The individual prevalence based on animals condemned at the abattoirs in relation to total animals slaughtered was similarly high oscillating at 14% (95% CI=2.4-26.4%). While the disease has been persistently reported for some time now [12,24,27,32], it seems to have either remained stable or actually spreading which may be explained by risk factors contributing to its sustenance coupled with the lack of enforcement of preventive and control measures in place. Due to the above observations, this particular study also tried to investigate factors related to either spread, sustenance as well as other risk factors associated with bTB positivity among cattle herds being high in Namwala District of Zambia. The univariate analysis incriminatingly pointed at a broad range of factors as being explanatory to bTB transmission in the Namwala District of Zambia. One of the major factors observed for the high bTB prevalence in Namwala District was related to the old system of traditional livestock keeping, especially through the transhumance system. The transhumance system exposed cattle to the Lechwe antelopes, which have been identified as untreatable reservoir hosts of bTB occurrence in the Kafue flood plains. Other herd-level factors were high herd sizes. In Namwala District, some cattle owners owned several thousand herds of cattle, which are a risk factor for TB [7,27,33]. This was further complicated by the sharing of common-pool resources, such as grazing grounds and drinking water through large assemblages of various cattle herds. Such factors have contributed to the continuum of the bTB transmission intra and between herds. Other risk factors related to the possible occurrence of the disease in human populations. The results showed high frequency of respondents consuming non-pasteurized milk at 81%. Only 26% of those interviewed indicated boiling their milk before consumption. Further, drinking of sour milk was as high as 79% when considered as the main ration consumed at least once per week. Zeroing in on what may be sustaining the disease in the domestic animals, the level of knowledge of bTB among farmers was only 40%, and thus 60% of the people may not be employing precautionary measures against the disease even when the Government of Zambia is among countries employing test and slaughter method of TB control. The tuberculin skin test using the purified protein derivative is used.

It is well established that bTB has been known to affect humans for a long time. In fact, even to this day in spite of pasteurization and test and slaughter having been used successfully to bring down the incidence in the West, still, there are cases in countries such as the USA, especially in the binational communities [34]. This demonstrates the need for continued efforts to study the disease, and GIS is an added tool that can help [13,14].

In summary, when considered independently, some of the significant factors that showed high proportional representativeness as contributing to disease transmission and maintenance included the low level of knowledge on bTB at only 41%, (95% CI; 30.6-50.6) and the correspondingly high contact between domestic animals and wild animals at 92% (95% CI; 86.0-97.3). Other plausible factors for possible transmission to humans as a zoonotic risk included low number of those boiling their milk at only 26% (95% CI; 17.1-35.0) coupled with low level of BCG vaccination among children at 29% (95% CI 19.9-38.4%). The multiple logistic regression model identified contact with wildlife, being transhumant (thus moving of cattle to the plains for grazing for certain parts of the year), cattle sharing grazing ground and drinking water as well as having animals being condemned at the abattoir due to suspected TB in the herd as factors having significant effect on herd bTB status. The Hosmer-Lemeshow goodness-of-fit check showed that the model fitted the data well with n=96, *χ*^2^ (6)=5.2, and p=0.52. Cattle herds that were reported to be sharing grazing grounds and drinking water with wild animals concurrently had a significant effect on bTB status (OR=5.5, 95% CI; 4.1-73.4) given that contact with wildlife and having animals being condemned at the abattoir due to suspected TB are controlled. While there were no tests done to ascertain the occurrence of bTB in humans during the study, it can be assumed that some human cases were related to the disease originating in animals. The assumption is borne out by the fact that at least 65% of those interviewed had been treated for TB and had bTB-positive herds. BTB has been known to be transmissible through drinking water to animals and in turn can be transmitted to humans if unpasteurized milk or contaminated carcasses are consumed. The contact with wildlife, which also leads to disease transmission to domestic animals, was as high as 52% and the sharing of drinking water at 91.67% in those interviewed. Contact with wildlife seems to be the primary factor since wildlife has been known to harbor bTB. Moreover, Lechwe antelopes found in Lochinvar National Park are important reservoirs of the disease. The influence of contact is highlighted by the maps generated in the study which indicates the geographical areas where the majority of bTB cases are originating from. From these maps, it is evident, given the larger circle, indicating the relative space covered by positive herds occurring along the GMAs of the Lochinvar National Park, the sanctuary of the Lechwe antelopes. The occurrence of bTB was more in the herds closer to Lochinvar National Park in the eastern region than in the western part of Namwala.

Further data indicate that 70% of the respondents living within the buffer zones of the national parks indicated having lost an animal at the abattoir due to suspected tuberculous lesions. To test for association with the geographical area, a STATA command [35] for Moran’s I was used and yielded a p=0.006 indicating a strong correlation or clustering of bTB-positive herds being more likely to occur in the GMA of the Lochinvar National Park (eastern interface of Namwala District), than on the western interface area, where there were no Lechwe antelopes. Most bTB-positive herds were within buffer zones of national parks within a 30 km radius and the buffer of game management zones within a 10 km radius. Water points were the most important factor wherein multiple logistic regression model, there were 5.5 higher odds of bTB cases in cattle sharing water with wildlife than those not sharing water. The findings bear some similarities to the effect of winter housing on bTB in the United Kingdom, where overwintering of animals brings them to close proximity with each other, which is a similar effect to waterholes in Zambia [36,37]. The commonality stems from the close contact between animals, which enhances disease transmission since TB is a contagious disease of close proximity. The strong effect of the three main risk factors as determined on univariate analysis were contacted with wildlife being highly significant (p=0.002), movement to the plains was also highly significant (p=0.001) and sharing of drinking water (p=0.017) all of which can be summarized as the strong effect of mixing between wildlife and domestic animals.

While mapping is interested in finding patterns, no information was gathered on how synchronized bTB is in herds of Namwala as done in other studies [38]. With the limited impact of control programs where farmers do not implement measures of test and slaughter effectively, the disease tends not to be synchronized. Distribution tends to mirror different practices by farmers, grazing fields, proximity to wildlife sanctuaries, and watering points. The results, therefore, indicate a spatial distribution of bTB as being clustered and closer to Lochinvar National Park GMAs, as obtained through the application of GIS tools in public health.

Transmission of bTB from wild animals to cattle, which poses a threat to humans has been widely studied [39]. This study identified the populations at risk as those in the interface of national parks and GMAs. Understanding spatial distribution and locating affected herds can help tremendously in containing the prevalence of bTB. Such success has been noted in developed countries, including Scotland [36]. It is hoped that using GIS tools; similar success can be achieved in Zambia. In our present study, spatial aggregation of disease was not quantified, but we assessed spatial distribution in relation to factors associated with bTB positivity such as close proximity to wildlife sanctuaries, watering points, and grazing strategies as outlined above.

## Conclusion

Based on the results obtained, we conclude that the spatial distribution of TB in Namwala follows the interface area where the Lechwe antelopes overlap with cattle in terms of grazing ground and watering points as captured. More importantly, the results showed that the prevalence is much higher in the eastern region of the district. The eastern region also happens to be closer to Lochinvar National Park, an important habitat of Lechwe antelopes which are known to harbor bTB. Spatial distribution of bTB is thus amenable to the use of GIS. Moreover, GIS can complement traditional epidemiological study methods such as questionnaire surveys.

## Authors’ Contributions

NFPT conceptualized the aim of the study, design, field study, georeferencing, and development of the first draft of the manuscript. BH helped in the conceptualization of the study, supervision, and revision of the manuscript. JBM helped in the conceptualization of the study, supervision, and revision of the manuscript. MM helped with field studies, supervision of data analysis, and revision of the manuscript. All the authors have read and approved the final manuscript.

## References

[1] Ashford D.A, Whitney E, Raghunathan P, Cosivi O (2001). Epidemiology of selected mycobacteria that infect humans and other animals. Rev. Sci. Tech.

[2] Armbruster C (1994). Infections with *Mycobacterium tuberculosis* and MOTT (Mycobacteria other than tuberculosis) in HIV infected and AIDS patients. Wien. Med. Wochenschr.

[3] Vayr F, Martin-Blondel G, Savall F, Soulat J.M, Deffontaines G, Herin F (2018). Occupational exposure to human *Mycobacterium bovis* infection:A systematic review. PLoS Negl. Trop. Dis.

[4] Davidson J.A, Loutet M.G, O'Connor C, Kearns C, Smith R.M, Lalor M.K, Zenner D (2017). Epidemiology of *Mycobacterium bovis* disease in humans in England, Wales, and Northern Ireland, 2002-2014. Emerg. Infect. Dis.

[5] Elliott A.M, Luo N, Tembo G, Halwiindi B, Steenbergen G, Machiels L, Pobee J, Nunn P, Hayes R.J, McAdam K.P (1990). Impact of HIV on tuberculosis in Zambia:A cross-sectional study. BMJ.

[6] Gopalan N, Chandrasekaran P, Swaminathan S, Tripathy S (2016). Current trends and intricacies in the management of HIV-associated pulmonary tuberculosis. AIDS Res. Ther.

[7] Broughan J.M, Judge J, Ely E, Delahay R.J, Wilson G, Clifton-Hadley R.S, Goodchild A.V, Bishop H, Parry J.E, Downs S.H (2016). A review of risk factors for bovine tuberculosis infection in cattle in the UK and Ireland. Epidemiol. Infect.

[8] Kharsany A.B.M, Karim Q.A (2016). HIV Infection and AIDS in Sub-Saharan Africa:Current status, challenges and opportunities. Open AIDS J.

[9] Cook A.J, Tuchili L.M, Buve A, Foster S.D, Godfrey-Fausett P, Pandey G.S, McAdam K.P (1996). Human and bovine tuberculosis in the Monze district of Zambia--a cross-sectional study. Br. Vet. J.

[10] Sarris A (2001). The Role of Agriculture in Economic Development and Poverty Reduction :An Empirical and Conceptual Foundation.

[11] Schneider M.C, Machado G (2018). Environmental and socioeconomic drivers in infectious disease. Lancet Planet. Health.

[12] Malama S, Johansen T.B, Muma J.B, Munyeme M, Mbulo G, Muwonge A, Djønne B, Godfroid J (2014). Characterization of *Mycobacterium bovis* from humans and cattle in Namwala district, Zambia. Vet. Med. Int.

[13] Fletcher-Lartey S.M, Caprarelli G (2016). Application of GIS technology in public health:Successes and challenges. Parasitology.

[14] Gemperli A, Sogoba N, Fondjo E, Mabaso M, Bagayoko M, Briët O.J, Anderegg D, Liebe J, Smith T, Vounatsou P (2006). Mapping malaria transmission in West and Central Africa. Trop. Med. Int. Health.

[15] Carney T.J, Shea C.M (2017). Informatics metrics and measures for a smart public health systems approach:Information science perspective. Comput. Math. Methods Med.

[16] Cudahy P.G.T, Andrews J.R, Bilinski A, Dowdy D.W, Mathema B, Menzies N.A, Salomon J.A, Shrestha S, Cohen T (2019). Spatially targeted screening to reduce tuberculosis transmission in high-incidence settings. Lancet Infect. Dis.

[17] Tanser F.C, Le Sueur D (2002). The application of geographical information systems to important public health problems in Africa. Int. J. Health Geogr.

[18] Torres-Gonzalez P, Cervera-Hernandez M.E, Martinez-Gamboa A, Garcia-Garcia L, Cruz-Hervert L.P, Bobadilla-Del Valle M, Ponce-de Leon A, Sifuentes-Osornio J (2016). Human tuberculosis caused by *Mycobacterium bovis*:A retrospective comparison with *Mycobacterium tuberculosis* in a Mexican tertiary care centre, 2000-2015. BMC Infect. Dis.

[19] Noble D, Smith D, Mathur R, Robson J, Greenhalgh T (2012). Feasibility study of geospatial mapping of chronic disease risk to inform public health commissioning. BMJ Open.

[20] Pigott D.M, Howes R.E, Wiebe A, Battle K.E, Golding N, Gething P.W, Dowell S.F, Farag T.H, Garcia A.J, Kimball A.M, Krause L.K, Smith C.H, Brooker S.J, Kyu H.H, Vos T, Murray C.J, Moyes C.L, Hay S.I (2015). Prioritising infectious disease mapping. PLoS Negl. Trop. Dis.

[21] Shaw N, McGuire S (2017). Understanding the use of geographical information systems (GIS) in health informatics research:A review. J. Innov. Health Inform.

[22] Shaweno D, Karmakar M, Alene K.A, Ragonnet R, Clements A.C.A, Trauer J.M, Denholm J.T, McBryde E.S (2018). Methods used in the spatial analysis of tuberculosis epidemiology:A systematic review. BMC Med.

[23] Pfeiffer D.U, Robinson T.P, Stevenson M, Stevens K.M, Rogers D, Clements A.C.A (2008). Spatial Analysis in Epidemiology. Oxford University Press, Oxford.

[24] Malama S, Muma J.B, Godfroid J (2013). A review of tuberculosis at the wildlife-livestock-human interface in Zambia. Infect. Dis. Poverty.

[25] Lamichhane B.R, Persoon G.A, Leirs H, Poudel S, Subedi N, Pokheral C.P, Bhattarai S, Gotame P, Mishra R, de Iongh H.H (2019). Contribution of buffer zone programs to reduce human-wildlife impacts:The case of the Chitwan National park, Nepal. Hum. Ecol.

[26] Munyeme M, Rigouts L, Shamputa I.C, Muma J.B, Tryland M, Skjerve E, Djønne B (2009). Isolation and characterization of *Mycobacterium bovis* strains from indigenous Zambian cattle using spacer oligonucleotide typing technique. BMC Microbiol.

[27] Munyeme M, Munang'andu H.M (2011). A review of bovine tuberculosis in the Kafue basin ecosystem. Vet. Med. Int 2011:Article ID 91∧.

[28] Zieger U, Pandey G.S, Kriek N.P, Cauldwell A.E (1998). Tuberculosis in Kafue lechwe (*Kobus leche kafuensis*) and in a bushbuck (*Tragelaphus scriptus*) on a game ranch in central province, Zambia :Case report. J. S. Afr. Vet. Assoc.

[29] Marianelli C, Amato B, Boniotti M.B, Vitale M, Ciarello F.P, Pacciarini M.L, Di Marco Lo Presti V (2019). Genotype diversity and distribution of *Mycobacterium bovis* from livestock in a small, high-risk area in northeastern Sicily, Italy. PLoS Negl. Trop. Dis.

[30] Palisson A, Courcoul A, Durand B (2016). Role of cattle movements in bovine tuberculosis spread in France between 2005 and 2014. PLoS One.

[31] Ghebremariam M.K, Rutten V.P.M, Vernooij J.C.M, Uqbazghi V, Tesfaalem T, Butsuamlak T, Idris A.M, Nielen M, Michel A.L (2016). Prevalence and risk factors of bovine tuberculosis in dairy cattle in Eritrea. BMC Vet. Res.

[32] Orloski K, Robbe-Austerman S, Stuber T, Hench B, Schoenbaum M (2018). Whole-genome sequencing of *Mycobacterium bovis* isolated from livestock in the United States, 1989-2018. Front. Vet. Sci.

[33] Nugent G, Gortazar C, Knowles G (2015). The epidemiology of *Mycobacterium bovis* in wild deer and feral pigs and their roles in the establishment and spread of bovine tuberculosis in New Zealand wildlife. N. Z. Vet. J.

[34] Gallivan M, Shah N, Flood J (2015). Epidemiology of human *Mycobacterium bovis* disease, California, USA, 2003-2011. Emerg. Infect. Dis.

[35] Bruin J (2006). New Test:Command to Compute New Test. UCLA:Statistical Consulting Group.

[36] Moustakas A, Evans M.R (2015). Coupling models of cattle and farms with models of badgers for predicting the dynamics of bovine tuberculosis (TB). Stoch. Environ. Res. Risk Assess.

[37] Moustakas A, Evans M.R (2016). Regional and temporal characteristics of bovine tuberculosis of cattle in Great Britain. Stoch. Environ. Res. Risk Assess.

[38] Moustakas A, Evans M.R (2018). Abrupt events and population synchrony in the dynamics of bovine tuberculosis. Nat. Commun.

[39] Moustakas A, Evans M.R (2017). A big-data spatial, temporal and network analysis of bovine tuberculosis between wildlife (badgers) and cattle. Stoch. Environ. Res. Risk Assess.

